# Prognosis and treatment of 46 Chinese pediatric cystic fibrosis patients

**DOI:** 10.1186/s12887-021-02789-8

**Published:** 2021-07-28

**Authors:** Qionghua Chen, Yuelin Shen, Hui Xu, Xiaolei Tang, Haiming Yang, Shunying Zhao

**Affiliations:** 1 Department No. 2 of Respiratory Medicine, Beijing Children’s Hospital, Capital Medical University, National Center for Children’s Health, Beijing, 100045 China; 2 Beijing Children’s Hospital, Capital Medical University, National Center for Children’s Health, 56 Nanlishi Road, Xicheng District, Beijing, 100045 People’s Republic of China

**Keywords:** Cystic fibrosis, Follow-up, Chinese children

## Abstract

**Background:**

Since public awareness of cystic fibrosis (CF) has increased, more children have been diagnosed with CF in China. This study aimed to investigate medical and other challenges faced by pediatric CF patients in China.

**Method:**

Treatments and treatment outcomes were retrospectively analyzed for 46 pediatric CF patients diagnosed from August 2009 to June 2019. Pre- and post-treatment results were compared using independent samples t-test.

**Results:**

Of 46 pediatric CF study patients, four died and five were lost to follow-up. Thirty-seven patients were monitored for 0.03 to 9.21 years; patients exhibited fewer attacks of respiratory tract infections after diagnosis (4.49 ± 2.13 episodes/year before diagnosis vs 1.97 ± 1.87 times/year after 1-year treatment, *p* < 0.05), significantly reduced sputum production and experienced 1.62 ± 1.71 exacerbations/year. Patient mean body mass index was 16.87 ± 3.53 and pancreatic malfunction persisted in 15 patients. For 17 children, no significant differences in lung function were found at follow-up as compared to lung function at diagnosis (FEV_1_: 82.45% ± 16.56% vs 75.26% ± 22.34%, FVC: 87.18% ± 13.64% vs 86.99% ± 19.95%, FEF_75%_: 46.51% ± 28.78% vs 36.63% ± 24.30%, *P* = 0.27, 0.97, 0.20, respectively). *Pseudomonas aeruginosa* (17/27) and bronchiectasis (22/22) were found during follow-up evaluation. Twenty-four patients (64.8%) maintained good adherence to therapies. Overall, azithromycin and tobramycin treatments were administered for 0.5–62 months and 0.5–48 months, respectively, and triggered no obvious adverse reactions.

**Conclusion:**

No obvious declines in clinical presentation or lung function were found in Chinese pediatric CF patients after receiving standard therapeutic and active treatments, although malnutrition and low compliance were persistent challenges.

**Supplementary Information:**

The online version contains supplementary material available at 10.1186/s12887-021-02789-8.

## Introduction

Cystic fibrosis (CF) is a common life-limiting autosomal recessive genetic disorder, with highest prevalence in Europe, North America and Australia. The disease is caused by mutations within the *CFTR* gene that encodes a chloride-conducting transmembrane channel, the cystic fibrosis transmembrane conductance regulator (CFTR), which regulates anion transport and mucociliary clearance within airways [1]. CF can cause abnormalities in respiratory, digestive, endocrine and reproductive systems, with patient prognosis depending largely on the extent of lung involvement. Early nutritional intervention and monitoring to detect respiratory and gastrointestinal disease in infants with CF are vital for improving long-term outcomes [2]. Since 1938, when cystic fibrosis was first identified, more and more cases have been reported worldwide [3]. However, epidemiological reporting of incidence rates has been limited in China until recently [4]. Indeed, only about 113 Chinese cases of CF have been reported [5], of whom most were diagnosed only within the past few years. The median age at CF diagnosis is currently 8.7 years in Chinese patients, according to results of a systematic review report [5]. This median age at diagnosis is significantly delayed as compared to the median age at diagnosis of 0.5 years reported in European patients [6] and of 3 months reported in American patients (according to the 2017 Patient Registry Annual Data Report). Here, factors underlying CF patient diagnostic delays and obstacles patients face in maintaining treatment compliance and achieving positive outcomes were investigated by retrospectively reviewing patient records in order to better understand challenges faced by children with cystic fibrosis in China.

## Subjects and methods

### Patients

Forty-six children diagnosed with CF at the Second Department of Respiratory Medicine of Beijing Children’s Hospital affiliated with Capital Medical University from August 2009 to June 2019 were included. A diagnosis of cystic fibrosis was mainly based on clinical manifestations, family history, positive sweat test and/or gene mutations of *CFTR* [7]. Informed consent was obtained from parents and/or legal guardians of the participants during follow-up visits. The study was performed in accordance with the Declaration of Helsinki and approved by the Institutional Review Board of Beijing Children’s Hospital, Capital Medical University, National Center for Children’s Health (number [2021]-E-034-R).

### Methods

Clinical data were retrospectively analyzed and assessed. Variables of age, gender, onset age, age at diagnosis, presence of mutation, clinical manifestations, body mass index, lung function, bacterial colonies, complications, treatments and patient outcomes were recorded.

#### Sweat conductive test

The Macroduct collection system and Sweat-Chek conductivity analyzer (Wescor Inc., Logan, Utah, USA) were used for CF sweat conductivity testing and analysis. Based on procedures in the user’s manual, sweat secretion was stimulated by pilocarpine iontophoresis. Following stimulation, sweat was collected for 30 min via coiled plastic tubing emptying into a collector cup then the sweat sample was transferred from the Macroduct tube to the take-up tube located on the conductivity cell then subjected to conductivity analysis. The test was repeated on 2 separate days then average results were calculated and considered normal if values fell below 60 mmol/L and intermediate if values were between 60 and 80 mmol/L. A diagnosis of CF was very likely for cases with values equal to or exceeding 80 mmol/L [8].

#### Analysis of CFTR genetic mutation

DNA samples were extracted from peripheral blood leukocytes using standard genomic DNA purification methods. Whole-exome sequencing, bioinformatics analysis and Sanger sequencing validation were performed according to standard protocols [9]. Multiplex ligation-dependent probe amplification was employed to detect large deletions or duplications of the *CFTR* gene.

#### Assessment of adherence

We provided regular follow-up for patients every 3 months to confirm that they were following doctors’ recommendations in addition to two or more clinical appointments per year. Patient compliance with doctors’ prescriptions and engagement in regular follow-up visits were considered evidence of good patient adherence to recommended therapies.

### Statistical analysis

Quantitative data were presented as the Mean ± SD for continuous values; numbers (%) for categorical values and non-parametric data were presented as median and extreme values. To compare results obtained between the two groups, we analyzed data using independent samples t-tests, with a value of *P* < 0.05 considered statistically significant.

## Results

### Characteristics of patients

Patient characteristics at initial assessment are shown in Tables 1. Of 46 pediatric cystic fibrosis patients, 5 were diagnosed based on Clinical + sweat chloride results, 33 were diagnosed by Clinical + sweat chloride + genetic mutation results and 8 were diagnosed based on Clinical + genetic mutation results. Overall, the mean age of onset of symptoms was 4.05 ± 3.77 years (ranging from 0 to 12 years old) and mean age at diagnosis was 8.05 ± 4.26 years (ranging from 0.59 to 15.88 years). A 4.01-year diagnostic delay on average was found between the time of first clinical presentation to when the patient received a definitive diagnosis. Fifty-one *CFTR* mutations were detected, with c.2909G > A and c.1000C > T detected most frequently (5 cases) and one child found to be homozygous for c.1521-1523delCTT (F508del). Thirty-six children harbored 2 mutations and 2 children harbored 3 mutations. Overall, six cases with mutations were homozygotes, 32 cases were compound heterozygotes and 3 cases had one CFTR allele mutation. Respiratory symptoms included persistent coughing (46/46), wheezing (12/46) and hemoptysis (4/46). Other main manifestations included pancreatic insufficiency, sinusitis, allergic bronchopulmonary aspergillosis [11], asthma and malnutrition. For 3 children, chief symptoms at CF onset included poor feeding, wasting, diarrhea, abdominal pain, weight stagnation or rectal prolapse. Thirty-eight children completed sweat conductive tests and had values ranging from 45 to 168 mmol/l (108.55 ± 25.68 mmol/l). Thirty-one children over 6 years of age completed lung functional assessments, with 19 cases showing different degrees of obstruction. Meanwhile, most patients (33/46) harbored *Pseudomonas aeruginosa* (*Pa*) infections that were detected at first admission, with 5 cases effectively clearing *Pa* and 14 failing to clear *Pa* during treatment. Other organisms isolated from study patients included filamentous fungi, *Candida albicans*, *Klebsiella pneumoniae*, *Haemophilus influenzae Stenotrophomonas maltophilia*, *Streptococcus viridis*, *Achromobacter* spp., *Geomyces* spp., *Aspergillus* spp. (including *A. fumigatus, A. niger*, *A. flavus)* and *Acinetobacter* spp. (including *A. baumannii*, *A. junii)*.

**Table 1:** Clinical features of 46 Chinese CF patients at the time of diagnosis

Variables	***N*** = 46
**Sex –no (%)**	46
Female - no (%)	20 (43.48%)
Male - no (%)	26 (56.52%)
**Age of onset of symptoms -year**	4.05 ± 3.77 (0–12, median2.50)
**Age at diagnosis -year**	8.05 ± 4.26 (0.59–15.88, median 8.74)
**Diagnostic delay -year**	4.01 ± 3.72 (0.14–12.44, median 3.05)
**Pulmonary symptoms**	
Cough -no/total no (%)	46/46 (100.00)
Wheezing -no/total no (%)	12/46 (26.09)
**Extra pulmonary symptoms**	3/46 (6.52)
**Respiratory infection times**	4.49 ± 2.13
**Pancreatic malfunction –no/total no (%)**	18/46 (39.13)
**ABPA -no/total no (%)**	14/46 (30.43)
**Asthma -no/total no (%)**	10/46 (21.74)
**Lung function** -no/total no (%)	31/46 (67.39)
FEV1	75.44% ± 22.40% (25.1–118.2%,74.5%)
FVC	83.59% ± 16.99% (33.1–113.9%,86.1%)
FEF75	39.45% ± 26.68%(6.6–112.9%,33.3%)
**Bacterial colonies n (%)** -no/total no (%)	46/46 (100.00)
PA -no/total no (%)	33/46 (71.74)
SA -no/total no (%)	9/46 (19.57)
**CT scan**	46/46 (100.00)
Bronchiectasis -no/total no (%)	42/46 (91.30)
Only Inflammation -no/total no (%)	4/46 (8.70)
Mucus plugging -no/total no (%)	14/46 (30.43)
Pleural thickening -no/total no (%)	14/46 (30.43)

Plus–minus values are means ± SD.

*ABPA* Allergic bronchopulmonary aspergillosis, *PA Pseudomonas aeruginosa*, *SA Staphylococcus aureus*. Normal/mild disease: FEV1 > 70% predicted, Moderate obstruction: FEV1 ranges from 40 to 69% predicted, Severe disease: FEV1 less than 40% [10].

### Treatment measures

Management of long-term CF treatment included daily inhalation therapy of hypertonic saline, intermittent inhalation of tobramycin, azithromycin treatment, airway clearance, chest physiotherapy and nutritional support. For mild pseudomonas exacerbation, we administered oral fluoroquinolone. For moderate or severe pseudomonas exacerbation, or in the case of failure of the above mentioned regimen, we treated patients with an antibiotic combination treatment containing cefoperazone sulbactam, ceftazidime and imipenem with cilastatin, or meropenem plus intravenous fluoroquinolone.

Details of long-term administration of a regimen consisting of azithromycin and tobramycin to the 37 study subjects were shown in Table 2. Azithromycin was administered orally for 3 days per week [12],at a dosage of 10 mg/kg per day. Three children were not prescribed azithromycin due to their mild CF symptoms; at follow-up, two of them lacked *Pa* infection and *Pa* had been eradicated in the third. No obvious side effects of treatment were observed, except for one case with elevated liver enzymes and 2 cases with abdominal pain that was relieved after short-term azithromycin cessation. With regard to tobramycin, injected tobramycin was used here in place of the nebulized form since the nebulized form was not available in China. For children over 6 years of age, a 300-mg dose was administered twice daily; for younger children, we instead used a dose of 160 mg. Tobramycin treatment alternated between 28-day administration and 28-day cessation.

**Table 2:** Long term regimen of Azithromycin and Tobramycin in stable phase of 37 children

Variables	***N*** = 37
**Azithromycin** -no/total no (%)	34/37 (91.89)
Number of actual users	31/34 (91.18)
Duration -month	25.69 ± 16.78 (0.5–62, median 23)
Onset time -month	3.77 ± 2.92 (1–12, median 3.0)
Side effect -no/total no (%)	3/34 (8.82)
**Tobramycin** -no/total no (%)	31/37 (83.78)
Number of actual users -no/total no (%)	29/31 (93.55)
Duration -month	24.60 ± 13.40 (0.5–48, median 24)
Side effect -no/total no (%)	0/31 (0.00)

With regard to airway management, the lack of professional physiotherapists in China has led to assignment of CF treatment management responsibilities to respiratory doctors, nurses and parents. With regard to nutrition support, generally all individuals with diagnosed CF received dietary recommendations to help them maintain adequate energy (high calorie and high protein) intake [13],. Meanwhile, it was also necessary to monitor children for emerging pancreatic insufficiency or inadequate replacement therapy if body growth trends were inadequate by administering lipase with meals as needed. In such cases, the starting dose for children younger than 4 years of age was 1000 lipase units/kg body weight per meal, with half of this starting dose administered to children older than 4 years of age at diagnosis. In addition, all children receive smaller lipase doses with high-fat between-meal snacks. Those patients experiencing symptoms of pancreatic insufficiency receive higher PERT doses that could reach a maximum of 2500 lipase units/kg body weight per meal. Fat-soluble vitamins and minerals (especially calcium) were given to all patients [14], while it was recommended that patients receive salt replacement when experiencing pulmonary exacerbation, gastroenteritis, heat stress or engaging in physically strenuous activities. Moreover, nutritional guidance has just begun to be provided in China and multi-disciplinary outpatient guidance is still extremely limited. Nevertheless, our follow-up evaluations revealed that 20 children could tolerate daily postural drainage, manual chest percussion and vibration-based measures provided by our current system of caregivers.

### Treatment adherence

Most of our pediatric CF patients resided in rural areas, attained a low level of education and belonged to families shouldering heavy economic burdens. Their parents struggled to administer long-term treatments (fraught with side effects) to their children. Thus, poor adherence to recommended airway clearance measures, poor nutrition and irregular medication administration were frequently observed. However, 24 of our cases strictly complied with doctors’ prescriptions and regular follow-up visits. Meanwhile, 9 other patients once stopped aerosolized therapy or azithromycin, 3 others stopped taking all medicines for 1 year and 1 patient stopped for 2 years, all without aggravating their symptoms.

### Clinical outcomes

Forty-six children were monitored for more than 0.03 to 9.21 years (2.88 ± 1.72 years). Generally, patients maintained irregular hospital visits, with intervals of 0.5 to 12 months between visits or between exacerbations. Of the 46 total cases, 4 children died (3 girls and 1 boy) and 5 cases were lost to follow-up.

#### Fatal cases

One patient who died of a pulmonary infection had provided a sputum sample at the time of the first hospital visit that produced positive *Pa* culture results, while another patient died of respiratory failure attributed to a diagnosed infection with *Pa* and aspergillosis. The parents of the other two fatal cases were reluctant to reveal causes of death. These patients were seriously ill at time of diagnosis; all presented with diffuse bronchiectasis, while two cases had digestive system involvement and two cases had histories of irregular treatment compliance. The four patients who died, at 8, 12, 13 and 14 years of age, possessed respective genotypes of c.263 T > G, c.2909G > A/c.3196C > A, c.3G > A/c.1572C > A and c.374 T > C.

#### Patient survival

All 37 surviving children presented with the symptom of mild cough that was most evident in autumn and winter and during early morning and/or night. During treatment, attacks of respiratory tract infections were reduced from (4.49 ± 2.13)/year per patient to (1.97 ± 1.87)/year per patient (*P* < 0.05), as were severities of coughing and sputum production. However, these patients still experienced 1.62 ± 1.71 episodes of exacerbation per year that required oral, inhaled or intravenous antibiotics over the study follow-up period, although the severities of attacks in most children were lower than in non-survivors. Notably, 3 cases remained basically stable without additional treatment. Of the 28 children over 6 years of age, 17 (60.71%) completed lung function tests during follow-up, with normal results obtained for 9 children, while obstructions were found in the others, including 3 children with mild, three with moderate and 2 with severe obstructions. Nevertheless, after comparing follow-up results with results at diagnosis, no significant differences were observed in lung function (FEV_1_: 82.45% ± 16.56% vs 75.26% ± 22.34%, FVC: 87.18% ± 13.64% vs 86.99% ± 19.95%, FEF_75%:_ 46.51% ± 28.78% vs 36.63% ± 24.30%, *P* = 0.27, 0.97, 0.20 respectively). Meanwhile, of 27 (72.97%) patients who provided sputum samples, 17 were infected with *Pa* and 6 harbored *Staphylococcus aureus* in airways during acute attacks. After treatment, *Pa* infection persisted in 14 cases, with new emergence detected in 3 cases and elimination of *Pa* in 5 cases. Patient mean body mass index was 16.87 ± 3.54 (ranging from 12.74 to 26.12), while pancreatic malfunction persisted in 15 children. Lung CT scans of 22 survivors revealed increased bronchiectasis in 6 cases after treatment for 2 months to 5 years, decreased bronchiectasis in 3 cases after treatment for 2 months to 8 years and no change in bronchiectasis in 13 cases after treatment for 5 months to 5 years. Ultimately, all 37 surviving children were able to tolerate mild physical exertion, with 33 of them even able to attend school. However, as compared to the 24 patients with good compliance, 4 patients with poor compliance did not experience worse clinical manifestations in our study. Assessments of all 37 children at follow-up after they received long-term treatment are shown in Table 3.

**Table 3:** Follow up assessment of the 37 children after long term treatment

Variables	N = 37
**Follow up duration** -year	2.88 ± 1.72 (0.03–9.21, median 2.83)
**Age at follow up**	10.63 ± 5.23 (2.45–20.04, median 11.16)
**Pancreatic malfunction** -no/total no (%)	15/37 (40.54)
**BMI**	16.87 ± 3.54
**BMI centiles**	32.26% ± 32.75% (0.1–98.5%, 18.9%)
**BMI Z-score**	−0.78 ± 1.46
**Acute exacerbation times**	1.62 ± 1.71
**Respiratory infection times after treatment**	1.97 ± 1.87
**Lung function** -no/total no (%)	17/37 (45.95)
FEV1	75.26% ± 22.34% (34.8–120.2%, 80%)
FVC	86.99% ± 19.95% (41.4–116.5%, 92%)
FEF75	36.63% ± 24.30% (7–99.2%, 30.3%)
Change of lung function –no/total no (%)	17/37 (45.95)
Better -no/total no (%)	7/17 (41.18)
Worse -no/total no (%)	10/17 (58.82)
**Bacterial colonies -no/total no (%)**	27/37 (72.97)
PA -no/total no (%)	17/27 (62.96)
SA -no/total no (%)	6/27 (22.22)
**Changes of bronchiectasis -no/total no (%)**	22/37 (59.46)
Decrease -no/total no (%)	3/22 (13.64)
Unchanged -no/total no (%)	13/22 (59.09)
Increase -no/total no (%)	6/22 (27.27)

Plus–minus values are means ±SD

## Discussion

Our study analyzed treatment outcomes for a cohort of 46 Chinese children with CF who were monitored for 3 years on average. Generally, clinical phenotypes and the genotypic spectrum associated with Chinese pediatric CF cases differ from corresponding aspects of Caucasian cases [15]. As compared to results of previous studies demonstrating diagnostic delays of 5.7 [15] and 8.7 years [5], here we found a 4.01-year diagnostic delay as evidence that underdiagnosis of CF patients still occurs in China. This issue is likely due to the lack of universal screening, whereby late diagnoses or misdiagnoses delay treatment initiation and delivery that can adversely impact patient prognosis [16]. In one study of patients with delayed diagnoses, health of CF patients at time of diagnosis was significantly poorer than health of other patient groups due to their greater number of respiratory (*P* < .0001), gastrointestinal (*P* = .005) and failure-to-thrive manifestations (*P* < .0001), with adverse effects on CF patient growth and respiratory function outcomes observed (FEV1% and *P. aeruginosa* colonization) [17]. Due to the greater diversity of genotypes and phenotypes observed in Chinese CF cases as compared to Caucasian cases, diagnostic delay and poor adherence to therapy, treatment responses and prognoses of Chinese CF patients are likely more variable than for Caucasian CF patients. In addition, we should further investigate both the spectrum of CFTR gene mutations and the range of CF phenotypes in the Chinese population and apply what we learn toward improving CF education delivered to doctors and patients.

Antibiotics are indispensable for achieving control of chronic pulmonary infections and acute exacerbations in CF patients. In one study [18], annual FEV1% predicted rates of decline for 3 consecutive years were nearly 40% lower for patients using azithromycin as compared with matched controls. Azithromycin also reduced exacerbations and supported beneficial weight gain when administered for 6–12 months to CF patients in another open-label follow-up study [19]. In our study, overall azithromycin treatment for roughly 2-years after initiation at 3 months post-diagnosis (on average) did not trigger obvious adverse reactions or drug resistance. Moreover, intravenous tobramycin treatment during acute exacerbations had a beneficial effect on bacterial clearance rate and expectorate quantity in our study. However, additional studies are needed to evaluate intravenous tobramycin treatment safety in pediatric CF patients.

In this study, as in some previous studies [20, 21], treatment effectiveness has been undermined by low patient compliance with follow-up recommendations. Generally, treatment adherence of patients with chronic diseases rarely exceeds 80%, with adherence most often falling between 30 and 70% [22]. CF is a relentless disease that, even with complete adherence, leads to declining health of survivors that continues even into adulthood [23]. In addition, poor CF treatment adherence can lead to worse health outcomes and greater healthcare use and cost [24]. CF guidelines recommend that patients receive at least 4 clinical visits per year, lung function testing every 6 months and yearly culture-based microbiological testing of respiratory tract secretions. Each visit should include a routine physical examination, pulmonary tests and collection of sputum or cough swab cultures for microbiological assessment as part of a multi-disciplinary approach to care. In a 2017 report from Cystic Fibrosis Foundation, more than half of patients with CF complied with recommendations relating to clinical visits, respiratory culture-based testing and lung function testing. However, in other studies only 40% of patients met care guidelines [25]. Our patient compliance was still insufficient. Indeed, one study in France demonstrated that implementation of a tracking system significantly improved patient quarterly clinic visit attendance from 4.6 ± 2.3 in 2009 to 6.3 ± 4.6 in 2013 (*P* < 0.0001) as one solution for patient non-compliance [25]. Nevertheless, numerous obstacles to patient compliance exist, including long travel distances to health providers, financial issues, work and school conflicts and difficulties associated with clinical scheduling. Therefore, improved doctor-delivered patient education, regular follow-up reminders, specialized outpatient treatment and access to CF regional centers may be needed to improve patient compliance in China.

During follow-up we learned that 4 fatalities had occurred that have stemmed from severe illness, insufficient treatment adherence by some parents and the unavailability of pediatric lung transplantation in China for patients with chronic respiratory failure. Notably, 3 survivors remained stable without receiving long-term medication or daily airway cleaning that they might not have needed due to their mild disease severity. Conversely, CT findings in another 3 cases indicated worsening lung pathology at follow-up, although airway cleaning every day had led to reduced amounts of expectorated sputum and numbers of acute respiratory tract infections.

Pulmonary function testing is used to assess the severity of CF lung disease, with FEV_1_ testing especially useful for this purpose [10]. Failures of pediatric patients in completing pulmonary function testing were related to the lack of availability of such testing in some local hospitals and difficulties in completing such tests in very young patients. Regardless, no overall significant difference was observed between pulmonary function at diagnosis versus lung function at follow-up. However, improved pulmonary function observed in some patients were explained by timely treatment, shorter follow-up times or milder disease, emphasizing the fact that such testing should be used more frequently to monitor pulmonary function of the young CF patients. In fact, CT scans have been particularly valuable when used in combination with pulmonary function tests to monitor CF lung disease [26, 27]. Moreover, 13.64% of children exhibited improvement of bronchiectasis in our study that was attributed to effective treatment delivery. Nonetheless, routine pulmonary infection testing of pharyngeal swabs or collected sputa should be conducted by outpatient clinics in China in order to improve treatment delivery and clinical status.

Importantly, lack of nutritional intake has been a major weakness of CF management efforts in China. Because CF patients rarely receive guidance from nutritionists, parents must often adjust patient diets themselves. As is well known, BMIs of pediatric CF patients and adolescents aged 2–18 years should be greater than 50% of BMIs of healthy children of the same sex within the same age range [28]. However, only 11 children (29.72%) in this study received adequate nutrition, as recommended by accepted guidelines. Moreover, 10 children (27.03%) had BMIs lower than the fifth percentile of the same age group. These results reveal that malnutrition is a common and serious concern for pediatric CF patients in China that is tied to the lack of specialized nutritionists in China, lack of awareness by respiratory physicians of this issue, inadequate patient adherence [29], and poor patient living conditions. Factors that aggravate patient malnutrition include repeated chronic infections, discontinuation of drugs, poor compliance, pancreatic exocrine dysfunction and poor basic nutritional status. Most notably, the number of patients with pancreatic malfunction of the total number of CF patients was 18 of 46 (39.13%) at baseline and 15 of 37 (40.54%) at follow-up. The poor overall outcomes may be due to insufficient PERT dosages and/or poor recovery of pancreatic function. More specifically, in patients with persistent symptoms and signs of exocrine pancreatic insufficiency (EPI), patients prescribed appropriate dosages of PERT may still fail to respond to PERT treatment due to missed doses, improper storage of enzymes, expired enzymes, mistiming of PERT administration (taking PERT after meals), destruction of enteric coating and comorbidities (non–EPI-related conditions) [30]. Therefore, we advocate for inclusion of appropriate pancreatic enzyme replacement therapy within long-term treatment plans for these patients.

Our research had limitations. First, data were obtained from a single center and thus might not adequately represent all Chinese children with CF. Moreover, follow-up measures were not of sufficient quality or duration, while missing data prevented analysis of some variables. Nonetheless, our results highlight the need for improved CF patient quality of care and case management and should be confirmed in future investigations of larger patient cohorts to better understand challenges facing the Chinese CF population.

In conclusion, diverse CF severity, low patient compliance and insufficient long-term management by medical providers are challenges that CF patients currently face. Although we did not observe obvious deterioration of clinical status and lung function in our patients, malnutrition and low patient compliance should have received greater focus in this study and thus highlight the need for better systematic follow-up to improve pediatric CF patient management. We therefore recommend that greater efforts be made to deliver standardized and individual care management, strengthen education measures and provide comprehensive follow-up care beginning early in childhood to pediatric CF patients in China.

## Supplementary Information

**Additional file 1: Table S1.** Clinical features of 4 death cases.

**Additional file 2: Table S2.** Clinical data of 46 Chinese CF patients.

## Data Availability

The datasets used and analyzed during the current report are available from the corresponding author (Shunying Zhao) on request.
