# Supplementary material for: Prognosis and treatment of 46 Chinese pediatric cystic fibrosis patients
Source: BMC Pediatr. 2021 Jul 28;21:329. doi: 10.1186/s12887-021-02789-8 (PMC8317407; doi:10.1186/s12887-021-02789-8)
Supplement: Supplementary file 1 — Additional file 1: Table S1. Clinical features of 4 death cases. [file 12887_2021_2789_MOESM1_ESM.docx]

**Table S1 Clinical features of 4 death cases**

|  | Gender | Age at diagnosis  (year) | Age at death  (year) | Clinical presentation | Sweat test  (mmol/l) | CFTR gene mutation |
| --- | --- | --- | --- | --- | --- | --- |
| 1 | Female | 10 | 14 | Bronchiectasis/pancreatic malfunction/steatorrhea/ malnutrition | 105 | c.263T>G |
| 2 | Male | 0.17 | 13 | Bronchiectasis/sinusitis/ malnutrition | 108 | c.2909G>A; c.3196C>A |
| 3 | Female | 2 | 8 | Bronchiectasis/ pancreatic malfunction/bowel obstruction/rectal prolapse | 109 | c.3G>A; c.1572C>A |
| 4 | Female | 12 | 12 | Bronchiectasis/ sinusitis | 168 | c.374T>C |
